# Developing Pre-Implementation Strategies for a Co-Designed, Technology-Assisted Parenting Intervention Using the Consolidated Framework for Implementation Research (CFIR) and Expert Recommendations for Implementing Change (ERIC) Approach

**DOI:** 10.3390/ijerph21121599

**Published:** 2024-11-30

**Authors:** Grace Aldridge, Andrea Reupert, Ling Wu, Joshua Paolo Seguin, Patrick Olivier, Glenn Pringle, Marie B. H. Yap

**Affiliations:** 1 Turner Institute for Brain and Mental Health, School of Psychological Sciences, Monash University, Melbourne, VIC 3800, Australia; 2 School of Educational Psychology and Counselling, Monash University, Melbourne, VIC 3800, Australia; 3 Department of Human Centred Computing, Monash University, Melbourne, VIC 3800, Australia; 4 General Manager, Strategy and Growth, IPC Health, P.O. Box 171, Deer Park, VIC 3023, Australia; 5 Melbourne School of Population and Global Health, University of Melbourne, Melbourne, VIC 3800, Australia

**Keywords:** adverse childhood experiences (ACEs), parenting intervention, technology, co-design, implementation, consolidated framework for implementation research (CFIR), expert recommendations for implementing change (ERIC), community health service, research-to-practice gap

## Abstract

Background: Adverse childhood experiences (ACEs) are a major risk factor for mental disorders in children. Parenting interventions can mitigate the impact of family-level ACEs and subsequently improve young people’s mental health. However, a substantial research-to-practice gap hinders access to, and uptake of, available interventions. Aim: This study aimed to develop actionable strategies to support the implementation of an evidence-based, co-designed, technology-assisted parenting intervention by understanding potential barriers and facilitators from the perspectives of service providers working with families of children experiencing ACEs. Methods: We conducted one-on-one interviews with 14 staff at a community health service (six managers, eight service providers). A theoretical thematic analysis was used. The Consolidated Framework for Implementation Research (CFIR) guided the data collection and analysis of barriers and facilitators. Pre-implementation strategies were informed by The Expert Recommendations for Implementing Change (ERIC) compilation. The CFIR–ERIC matching tool was used to match the CFIR barriers identified by participants in this study with ERIC strategies to overcome these barriers. Results: Fourteen CFIR constructs were identified as facilitators, and eleven as barriers. By using the CFIR–ERIC tool, eleven strategies to mitigate the barriers were identified. Most strategies were aligned to the ERIC clusters Use evaluative and iterative strategies (*n* = 4) and Develop stakeholder interrelationships (*n* = 3). Conclusions: The CFIR–ERIC approach offered relevant and concise pre-implementation strategies for addressing potential barriers to implementing a novel, co-designed, technology-assisted parenting intervention for parents of children with ACEs. The identified facilitators support the utility of co-designing interventions as an initial phase in bridging research-to-practice gaps. Healthcare settings aiming to innovate services with technology-assisted parenting interventions to improve child mental health can draw on findings from the current study to guide pre-implementation plans for innovative, technology-assisted parenting interventions to improve child mental health.

## 1. Introduction

Depression and anxiety disorders are major sources of global disease burden for children and young people [1,2]. The highest prevalence rates of depression and anxiety disorders in Australia occur in young people aged 16–25 [3], which can significantly impact their health, social, and educational outcomes over the lifespan [4]. Exposure to adverse childhood experiences (ACEs), defined as stressful and potentially traumatic events during childhood or adolescence (e.g., child maltreatment and bullying), is strongly associated with the onset of mental disorders in young people including depression and anxiety [5,6]. Many systemic or societal-level ACEs are beyond an individual’s control, however, family-level ACEs, such as maladaptive parenting behaviours, are modifiable and an appropriate target for intervention [7].

Parenting interventions have demonstrated efficacy in reducing maladaptive parenting behaviours and sustainably improving child mental health outcomes such as depression and anxiety [8,9]. These interventions are typically delivered via community centres, within the family home, or in healthcare settings such as hospitals or health facilities [10]. Parents whose children experience ACEs also often experience increased physical and psychological barriers to engaging with parenting interventions [11,12]. Primary healthcare settings can potentially overcome physical and psychological barriers by offering population-level access, high frequency of family visits, potential for utilising existing infrastructure and resources, and opportunities to build on existing patient–provider relationships [13]. For this reason, they are especially well positioned to deliver universal preventive interventions.

Nonetheless, families may still face practical and cost-related barriers to accessing healthcare services (e.g., transportation challenges and limited time) [14], however, many of these barriers can be overcome with technology. The public health measures implemented to manage the COVID-19 pandemic necessitated an abrupt transition in health and social care from face-to-face delivery of services and interventions to telehealth delivery [15], highlighting the potential of technology in facilitating accessible child and family services. Moreover, technology-assisted interventions are easier to scale up and disseminate compared to face-to-face interventions [16]. Technology-assisted parenting interventions have been shown to be efficacious in improving parenting outcomes (including maladaptive parenting behaviours) and reducing child mental health problems [17,18,19] in the context of socio-economic disadvantage [20]. Prior research has also highlighted how the costliness and longstanding shortage of qualified practitioners has resulted in a need to find alternative methods of delivering interventions targeting family-level ACEs [21]. There is thus a compelling case for healthcare services to implement technology as part of delivering parenting interventions that aim to improve child mental health.

The time between developing innovative interventions and implementing them has historically been a long process, known as the research-to-practice gap [22]. For example, practitioners’ attitudes and skills towards technology-assisted innovations can also influence its implementation [23,24], and factors such as low compatibility between an innovation and existing services are known barriers to both implementing evidence-based parenting interventions and sustaining them in routine practice [25]. Research-to-practice gaps are especially common in services for children with ACEs [26], representing an additional barrier for families accessing evidence-based support.

Implementation science is an emerging field that broadly aims to improve the uptake of evidence-based practice in services and organisations by identifying and evaluating factors that may influence implementation, then developing strategies to overcome barriers and capitalise on potential facilitators [27]. Indeed, a common component of successfully disseminated interventions is the inclusion of a needs and barriers assessment to assess contextual factors (e.g., perceived value of intervention, resources) that may influence the integration and uptake of an innovation prior to implementation [28]. Given effective implementation is associated with improved target outcomes [29], prospectively assessing needs and barriers, and developing strategies to overcome barriers, represent critical steps in effectively implementing technology-assisted parenting interventions into services for parents of children with ACEs.

The clear and detailed reporting of implementation factors and strategies facilitates interpretation needs and barriers assessments, and, in turn, the synthesis of results across the literature [27]. The Consolidated Framework for Implementation Research (CFIR) is a widely used implementation science framework that supports clear and detailed reporting of implementation factors [30]. Developed through a synthesis of evidence on implementation factors shown to influence an innovation’s effectiveness, the CFIR provides a taxonomy of constructs to facilitate the systematic assessment and reporting of factors that may predict and/or explain barriers and facilitators to effective implementation of an innovation in a given setting [31]. To support clear and detailed reporting of implementation strategies, both prospectively and retrospectively, a compilation of implementation strategies was developed called the Expert Recommendations for Implementing Change (ERIC) [32]. Expert consensus on the most effective ERIC strategies for overcoming CFIR barriers and leveraging facilitators has resulted in the CFIR–ERIC matching tool, which is designed to facilitate effective strategy selection in implementation planning for healthcare services (see [33,34,35] for examples). However, a relative lack of consensus between experts regarding which strategies are most appropriate to address CFIR barriers means this tool should be used with caution [36]. Nonetheless, the aggregated list of expert-endorsed, actionable strategies is useful for prospectively planning to mitigate or overcome implementation barriers, and for supporting the systematic reporting and evaluations of implementation strategies. Efforts to minimise research-to-practice gaps in services for children with ACEs, and improve families’ access to evidence-based, technology-assisted parenting support, can thus be facilitated by using the CFIR–ERIC tool to prospectively assess a healthcare setting’s existing needs and barriers, and develop strategies to overcome barriers.

The aims of this study were two-fold: first, to understand potential barriers and facilitators from the perspectives of service providers working with families of children experiencing ACEs; and, second, to develop actionable strategies to support the implementation of an evidence-based, co-designed, technology-assisted parenting intervention.

## 2. Methods

### 2.1. Study Setting

This study was affiliated with a larger research program conducted by the Centre for Research Excellence in Childhood Adversity and Mental Health, and was conducted in partnership with one of the largest providers of community health services in Victoria operating across six sites in western Melbourne. Local Government Areas serviced by this community health service have culturally diverse populations that experience greater socio-economic disadvantage compared to Greater Metropolitan Melbourne [37].

### 2.2. Study Design

A descriptive qualitative research design [38] was used to answer this study’s aim. The publicly available CFIR Interview Guide Tool informed the semi-structured interview schedule [31]. The Tool recommends selecting and evaluating CFIR constructs of particular salience to the study’s context and stage of implementation [31], hence this study selected: intervention characteristics (relative advantage, adaptability), inner setting (implementation climate: compatibility, organisational incentives and rewards; readiness for implementation: leadership engagement, available resources), and characteristics of individuals (self-efficacy). Open-ended questions were also included to collect general feedback. See Supplementary Material for the semi-structured interview schedule. The Expert Recommendations for Implementing Change (ERIC) comprises 73 discrete strategies involving one action or process [32]. Level 1 strategies are those ranked by more than 50% of experts as one of their top seven strategies for a given CFIR barrier, and Level 2 strategies are those ranked by 20%–50% of experts as one of their top seven strategies [36]. This study used the CFIR–ERIC tool to identify pre-implementation strategies for overcoming CFIR barriers, and considered the two most highly-ranked ERIC strategies for addressing each CFIR barrier identified by participants.

### 2.3. Participants

Participants (*n* = 14) were staff from the community health service who work with parents of children aged 4–11 in a service provision or service management capacity. Purposive sampling was used to recruit either managers or service providers. All manager participants (*n* = 6) who were invited agreed to participate. All service provider participants (*n* = 8) who expressed interest were recruited and participated in the interviews (5/28 from Family Services, 1/19 from Child Health, and 2/8 from the Hub—a novel service integrating Family Services and Child Health in one place). No prior knowledge about the co-designed intervention was required of participants. Table 1 details participant demographics.

### 2.4. Recruitment and Study Process

Participants were recruited through services that work with families with children aged 4–11 (Family Services, Child Health, and the ‘Hub’—a new, central service co-locating services to support the mental health and wellbeing of children and families). The study was advertised through the staff intranet, targeted emails to eligible services’ managers, targeted emails from recruited managers to service providers within their service, and the researcher (GA) attending services’ team meetings to introduce and explain the study. All recruitment efforts involved the researcher stating their experience, training, research interests, and reasons for conducting the research. Participants expressed interest in the study directly to the researcher (GA) via email. Written consent was collected prior to the interview. All interviews took place during the staff’s rostered hours. Demographic information was collected after the qualitative interview via email and stored on the academic institution’s (Monash University) secure server in a password-protected folder. This study has been reported in line with the Consolidated Criteria for Reporting Qualitative Research [39] (see Supplementary Material).

### 2.5. The Evidence-Based, Co-Designed Hybrid Parenting Intervention: PaRK-Lite

PaRK-Lite is a technology-assisted parenting intervention co-designed with service providers from this study’s community health service. Its content is derived from the Parenting Resilient Kids (PaRK) intervention, a web-based parenting intervention designed to prevent or reduce internalising problems in children by targeting parental factors (including maladaptive parenting behaviours) associated with child internalising disorders, such as depression or anxiety [40]. PaRK is thus a highly appropriate intervention for services to offer in efforts to reduce or prevent maladaptive parenting as an ACE. PaRK’s core program content is derived from a set of guidelines on parenting strategies that evidence suggests can reduce or prevent a child from internalising problems (henceforth known as the Guidelines) [41]. The Guidelines were based on evidence from a systematic review [42] and Delphi expert consensus studies [43]. A randomised controlled trial of PaRK has shown improvements in parent-reported parenting behaviours that prevent a child from internalising problems, including maladaptive parenting behaviours [44].

The co-design process for adapting PaRK for the community health service has been reported in detail elsewhere [45]. In brief, it involved re-designing PaRK’s delivery format to better meet service providers’ needs and preferences for technology-assisted parenting programs in their service, and, in turn, facilitate its implementation. Needs included more simple technology and components to prompt parents’ reflection on their parenting, hence a hybrid intervention was co-designed, consisting of podcasts and micro-coaching sessions. PaRK was re-named ‘PaRK-Lite’ for the service to reflect the resulting lighter-touch approach to delivering PaRK content. Each podcast topic (*Helping your child manage emotions, Establishing rules and consequences,* and *Managing conflict in the home)* is comprised of 10× 3–5-min episodes that presents PaRK’s evidence-based content in a story-telling format representing everyday parenting challenges from the perspectives of parents, children, and extended family members. The brevity of episodes was designed to support parents engaging with the podcasts in ways that fit their schedules and pacing preferences. Micro-coaching sessions are held during existing service contacts between service providers and parents (either face-to-face or via telehealth), and focus on reviewing parents’ engagement with the podcasts and reflecting on the podcast content, towards empowering parents about changing parenting practices [46].

### 2.6. PaRK-Lite Presentation/Demonstration and Interview Content

Prior to the interview, participants viewed a video in their own time, which outlined PaRK-Lite’s evidence base, its co-design process with practitioners from Family Services, its intended benefits for the community health services working with families aged 4–11, and snippets of PaRK-Lite’s podcast and micro-coaching components.

Qualitative data were then collected via individual, semi-structured interviews using the above-described interview schedule (see ‘Study design’). The interview schedule was piloted with colleagues of the authors who have experience in conducting qualitative research with parents, and minor changes to simplify the wording of interview questions were made. Interviews focused on exploring participants’ perceived barriers to and facilitators of implementing PaRK-Lite in their service in a conversational manner along with probes that enabled participants to freely discuss and elaborate on their ideas and experiences [47].

### 2.7. Data Collection

All interviews (*n* = 14) were conducted between May and August 2023. Each interview was conducted by a female clinical psychology PhD Candidate (GA) with an interest in bridging research-to-practice gaps in innovative parenting interventions. GA also led PaRK-Lite’s co-design study [45], and hence had a prior relationship with two service provider participants and one manager participant from Family Services. Each interview was prefaced by GA restating the purpose of the interview in the context of the co-design approach and PaRK’s adaptation into PaRK-Lite, reiterating that any thoughts and feedback were valuable. Thirteen interviews were conducted via a secure web-conferencing platform (Zoom) and one interview was conducted face-to-face at the community health service. Only the researcher (GA) and interviewee were present during all interviews, and no repeat interviews were conducted. Interviews ranged from 40 min to 62 min, with a mean duration of 47 min. All interviews were audio recorded, and the interviewer made observational field notes during and after the interview to document in-the-moment reflections. All interviews were transcribed by a reputable third-party transcription service, and imported into NVivo to support data analysis. Data collection and preliminary analysis occurred concurrently.

### 2.8. Data Analysis

Participant data were tagged with an M or SP (depending on whether they were a manager or service provider participant, respectively) and an identifying number (e.g., SP1). Credibility of data was first achieved through member checking, in which participants are invited to check their interview transcript for accuracy and ensure it is resonant with their experience of the interview [48]; no participants returned their transcript with corrections or clarifications. During data immersion, interpretations emerging from transcripts were triangulated with field notes.

Interview data analysis was guided by recently published implementation science research that used the CFIR to inform the collection and analysis of implementation barriers and facilitators [49,50]. While the CFIR Interview Guide Tool that informed the semi-structured interview schedule is based on the original CFIR, the updated CFIR [51] informed the coding framework used to analyse data and identify barriers and facilitators discussed in the interviews. The updated CFIR comprises 48 constructs and 19 subconstructs divided into five domains: *innovation* (8 constructs); *outer setting* (7 constructs, 3 subconstructs); *inner setting* (10 constructs, 10 subconstructs); *individuals*: roles subdomain (9 constructs) and characteristics subdomain (4 constructs); and *implementation process* (8 constructs, 6 subconstructs). Only two constructs used in the semi-structured interview schedule from the original CFIR (*leadership engagement* from the *Inner setting* domain and *self-efficacy* from the *Characteristics of individuals* domain) were re-classified in the updated CFIR under the ‘*Individuals’* domain, hence data collected in response to these constructs were coded into this domain.

Using this as a framework, the first author then followed the six-steps process described by Braun and Clark [52], whereby she independently familiarised herself with, and subsequently analysed, all interview transcript data. Initial codes were generated using a start list informed by CFIR constructs included on the semi-structured interview schedule as pre-determined themes, with codes not relevant to the start list being mapped onto the relevant CFIR construct to develop the initial coding framework. Any differences in responses to the same construct between manager and service provider groups were explored, however none emerged. The framework was continually refined as data analysis progressed. Codes nested within each CFIR construct/theme were then re-coded to specify a barrier or facilitator. Initial barrier and facilitator codes were then reviewed by the interviewing researcher (GA) and the second author (AR), who held expertise in qualitative research and family intervention development and implementation, to improve credibility and generate initial themes. Themes were subsequently refined and defined by GA to produce the final coding framework. The second and third authors reviewed themes defined in the final coding framework, and any differences or disagreements were resolved through discussion prior to writing up the report.

The publicly available CFIR–ERIC matching tool tabulates each CFIR barrier and the 73 ERIC strategies with a percentage indicating the proportion of endorsement by implementation science and practice experts for each strategy to address each barrier [36]. In this study, the first two most highly-endorsed strategies were considered for each CFIR barrier. If any suggested strategies were not deemed applicable to the study-specific barrier by the author team, they were not included.

### 2.9. Ethics Approval

This study was approved by the Monash University Human Research Ethics Committee (2023/35024).

## 3. Results

**Figure 1:**
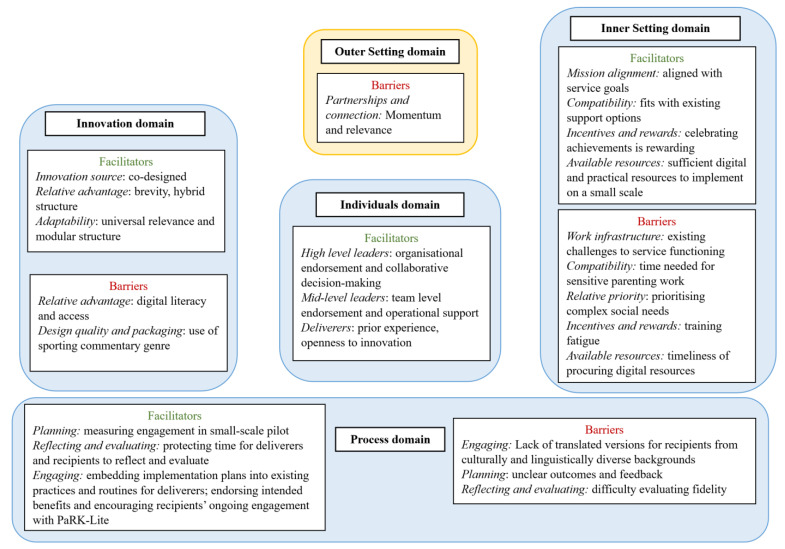
CFIR Barriers and Facilitators identified by participants.

Figure 1 summarises the facilitators and barriers in each CFIR 2.0 domain, as identified by participants and further detailed below. See Supplementary Material for further exemplar quotes for each barrier and facilitator.

### 3.1. Individuals Domain

The CFIR’s *Individuals Domain* construct captures the *roles*, *actions*, and *characteristics* of individuals involved in implementation.

### 3.2. Leaders

High-level leaders. Participants described how high-level leaders (such as the executive leadership team and service managers) could facilitate implementation by introducing PaRK-Lite’s intended purpose and benefits for the community health service, and endorsing it as a formal offering to model the leadership’s commitment to implementation, and fostering collaborative decision-making about how PaRK-Lite will be implemented and evaluated: “*the message coming top down is really important to encourage workers that it is worthwhile…that it’s supported and you’re supported to do it*.” (SP1).

Mid-level leaders. Participants indicated that mid-level leaders (such as team leaders) would likely play a significant role in facilitating implementation—“*the best operational leadership comes from someone who is part of the team*” (M15)—by introducing PaRK-Lite’s purpose and intended benefits specific to each team’s staff and client groups, providing ongoing encouragement and reminders about PaRK-Lite, and fostering collaborative problem-solving regarding PaRK-Lite’s delivery processes. Manager participants emphasised that team leaders would therefore need to be “*supported …on an organisational level to put the time aside*.” (SP9).

### 3.3. Intervention Deliverers

Participants discussed which staff were best placed to deliver PaRK-Lite based on their discipline, focus, and experience with coaching and family work, “*You want them to understand about good family-centred practice and good parenting principles.*” (M11). This included Family Services practitioners, psychologists, occupational therapists, and the Hub’s Family Support Workers. Staff who were identified as better placed to indirectly deliver PaRK-Lite through roles such as identifying and referring interested families, included Speech Pathologists and the Hub’s Wellbeing co-ordinators. Openness to and interest in innovation was also identified as a motivating factor for delivering PaRK-Lite, “*If there’s something new then I’m always willing to try it, and because I’m always looking for the best outcome for the client*.” (SP22).

### 3.4. Intervention Recipients

Participants reflected on characteristics of parents (i.e., the target recipients of PaRK-Lite) that would likely influence their engagement. Capability and interest with digital platforms were viewed as a critical factor for engagement, but that there may be “*another cohort [of parents or caregivers] that prefers that face-to-face, human contact and connection*” (M12) to engage with parenting support. Being on a waitlist and not yet receiving other interventions was viewed as a highly appropriate opportunity to receive PaRK-Lite: *“Why not offer them something like this while they’re waiting?*” (M15). However, as summarised by SP19, participants emphasised that “*parents absolutely need to be quite self-motivated in order to actively engage*”, with parent readiness for change and soft timelines for intervention completion being cited as factors that have historically supported motivation for ongoing engagement. PaRK-Lite’s flexible and empowering design was viewed as favourable for supporting parents in improving their parenting confidence and sense of control in otherwise unpredictable living circumstances, “*breaking it down can be a really great way of getting them to feel in control of at least one area*.” (SP10). However, the universal nature of PaRK-Lite’s content prompted some participants to query its relevance for parents of children with additional needs: “*We’re assuming these kids have no additional needs…but that’s going to impact on whether they’re going to be able to relate to what is given to them in these podcasts*...” (SP10).

### 3.5. Innovation Domain

The CFIR’s *Innovation Domain* construct captures constructs relating to the innovation being implemented, i.e., PaRK-Lite’s podcasts and micro-coaching design (see Section 2.5).

### 3.6. Innovation Domain Facilitators

Innovation source: Co-designed. PaRK-Lite being co-designed between researchers and service providers from the community health service was viewed favourably by participants with regards to its credibility. As illustrated by M13, “*You’ve certainly looked at all of the research … you’ve done all of the work as well as understanding the context*.”

Relative advantage: Brevity. The “*short, sharp*” (M13) modular format of PaRK-Lite was viewed as advantageous for engaging parents, compared to other lengthier, intensive parenting support options currently available through the community health service: “*I don’t really have a whole lot of short resources that are easy to deliver to parents*.” (SP17).

Relative advantage: Hybrid structure. PaRK-Lite’s hybrid structure consisting of a digital, self-guided learning component with a clinician-supported, individualised coaching component was viewed by participants as novel, with several advantages over other parenting supports available. SP9 described this advantage like “*building a bridge between that face-to-face service… to something that a family or a parent can access in their own time…that’s something that I hadn’t really seen before… it helps to integrate people’s learning…applying theory to practice*.” Compared to parenting support options which are entirely clinician-led, PaRK-Lite’s self-guided component was considered advantageous for empowering parents as it “*has the flexibility of working around what’s best for the client, which you don’t get with other programs*” and “*boost[ing] their confidence and self-esteem, that they can do it on their own*.” (SP1). Participants stated that the entertaining podcast format, which enables playback, would likely be advantageous for promoting parent learning because “*using a humorous way to break down that known barrier… was novel. It might just help to put people as ease*” (M12) and “*they can rewind and revisit with a bit more space in their own time versus being in a session and trying to be pushed along.*” (SP14). The individualised, micro-coaching component was advantageous in “*getting a more targeted and successful outcome [by] being authentic and client-focused*.” (SP14). One service provider elaborated that providing immediate parenting support with PaRK-Lite rather than referring to external parenting services would “*make my job very satisfying*” (SP16) by reducing parents’ frustration with waitlists.

Adaptability. Participants viewed PaRK-Lite as being adaptable due to its modular structure, (“the length of the podcast [and] length of the micro-coaching sessions could adjust up or down. The prompting questions in the templates could be altered as we [staff] learn.” (M15)), and the relevance of the podcast topics for parenting issues that participants across multiple services work on with parents. “When you look at family units trying to live together, those topics are often what come up.” (M11)

### 3.7. Innovation Domain Barriers

Relative (dis)advantage: Digital literacy and access. Participants reported that digital literacy and access is an ongoing barrier for parents in the Wyndham LGA due to socioeconomic disadvantage and internet outage issues known to the area: “*Families who are struggling with low socioeconomic… or maybe there’s a million reasons why they might not have access to that technology*.” (SP17).

Design quality and packaging: Use of sporting commentary genre. Participants expressed that, while the use of a sporting commentary genre to present the evidence-based information may be appealing to some families who like sports, it may also be a barrier “*for families who don’t have that in their culture…[they] might find the format maybe a bit confusing. They might not understand why they’re presenting in this commentator standpoint*.” (SP17).

### 3.8. Ideas to Adapt: PaRK-Lite’s Design and Delivery

Participants offered several ideas to enhance PaRK-Lite, including more pictures and visuals on the written artefacts (such as a schedule), building in an interactive component at the end of the podcast for parents to review their understanding, visuals or animations to accompany the podcasts for visual learners, creating a brochure or handout to explain PaRK-Lite’s purpose and intake procedures, and developing a fully self-guided online version of PaRK-Lite without micro-coaching. Participants also suggested leveraging podcasts’ entertaining genre to adapt PaRK-Lite for parent and child work, supporting the child-focused nature of their work. Several additional strategies were suggested to encourage parents’ uptake of PaRK-Lite, including offering PaRK-Lite as a step-down program after completing more intensive work and ensuring interpreters are available to support micro-coaching if translated evidence-based resources are provided.

### 3.9. Inner Setting Domain

The CFIR’s *Inner Setting Domain* construct captures constructs relating to the setting in which the innovation is being implemented, i.e., teams within the community health service that work with families of children aged 4–11.

### 3.10. Inner Setting Domain Facilitators

Mission alignment: Aligned with service goals. Participants in management roles discussed how PaRK-Lite’s co-design approach, cross-disciplinary applicability, digital format, and target recipients (children and families) are *“consistent with where the organisation is heading and its overall strategic objectives*.” (M12). Participants in service provider roles discussed how the micro-coaching’s parent-led approach were aligned with their perceived purpose to provide meaningful service experiences to clients. SP14 described this as follows: “*The overarching goal is the hopes for my child… you want that as the guiding journey or roadmap for why you’re participating in something. I think we do that with all of our services.*”

Compatibility: Fits with existing support options. PaRK-Lite was viewed as compatible with the existing suite of available parenting tools and resources currently used by service providers, and further diversified them: *“You can implement PaRK-Lite…then if PaRK-Lite doesn’t work, then you have another resource, and so on…until you meet the desired outcome... it’s great to have a diverse range of resources you can use to support families*” (SP17). Participants also discussed how PaRK-Lite would align, and sometimes enhance, their existing work processes and practices for individual client work.

The micro-coaching is the only bit we really need to do…that’s something we can incorporate into our work anyway. It’s very complementary to what we already do. (SP10)

If they’re interested in it, it’ll become part of the goal plan and then you implement it…as part of your casework with the family. (SP1)

Incentives and rewards: Celebrating achievements is rewarding. Participants discussed how noticing positive changes in parenting and child behaviour, and celebrating those achievements with parents, is a rewarding aspect of their role, and that the micro-coaching could facilitate that reward. “*It [micro-coaching] still keeps that activity happening. You’re still achieving things…I like to achieve things and I like to see my clients achieve things*.” (SP22).

Available resources: Sufficient digital and practical resources to implement on a small-scale. Participants reported that digital resources are overall much more abundant since COVID-19 lockdowns, which would likely facilitate implementing PaRK-Lite. “*Most people…even from different socio-economic backgrounds…usually have access to a smartphone…that in itself is obviously a good thing when it comes to reducing barriers*.” (SP19). Participants also reported that the practical resources to guide micro-coaching delivery *“was great because then that really saves us a lot of time of having to think about what we’re going to ask*” (SP22). Participants felt that a small-scale implementation would help illuminate whether further resources are required, given its novelty. As summarised by SP1, “*I think we have a lot of the resources…[but] It’s not something that we’ve done before in Family Services. So I think it will be one of those trial and errors on whether we’ve got the resources or not*.”

### 3.11. Inner Setting Domain Barriers

**Work infrastructure: Existing challenges to service functioning**. Full caseloads and long waitlists were viewed as existing challenges to providing “*core business*”, such that “*adding something more would be a stretch*” (SP14) and a barrier to delivering PaRK-Lite. As summarised by M15, “*It’s not about positive or negative intent, it is simply about how crowded their… attention space is for options when it comes to clients* (M15).” Some participants suggested a ‘key worker’ approach could overcome this, whereby a dedicated staff member delivers PaRK-Lite and acts as a link to other external parenting supports as needed: “*If it was someone’s role… then that would almost be nice, to know that someone is taking care of it—it wasn’t something else you needed to follow up*.” (SP8). Participants working in the Hub expressed that as the leadership team and practitioner team’s scope of practice is still being developed, “*to introduce something else on top of that now would probably feel a little bit overwhelming and a little bit complex.*” (SP8).

Compatibility: Time needed for sensitive parenting work. Participants reflected that discussions about parenting can lead to deeper conversations about factors influencing their parenting, which may be a barrier to feasibly fitting a 15-min micro-coaching session into existing service contacts. “*Parenting issues with families, those conversations typically can be quite lengthy…one issue could take up the whole session*.” (SP10)

Relative priority: Prioritising complex social needs. Participants discussed observations around how the social complexities families can present with (such as housing instability and risk of harm) can interfere with parents’ readiness to engage with parenting support. “*The barrier might be just if our client cohort had more complex needs*.” (M11).

Incentives and rewards: Training fatigue. Participants reported that the time needed for training and delivering PaRK-Lite could be a potential disincentive for staff to implement it due to the large volume of training taken in recent years and consistently full caseloads: *“Everyone is so busy and we’ve had to implement so many new things over the last few years…there’s a bit of that worker fatigue coming in…even though the program itself is quite simple*.” (SP1).

Available resources: Timeliness of procuring digital resources. Participants in service provision roles reflected that procuring digital resources for families (e.g., smart phones) requires managers’ approval and oversight, which was a barrier to the timely availability of such resources. “*I’m not in charge of that, so I’m not sure how viable that is*.” (SP17).

### 3.12. Ideas to Adapt: PaRK-Lite Training

Participants reflected on their experiences of prior training and identified several factors that would facilitate their engagement with PaRK-Lite training. This included covering PaRK-Lite’s evidence base (“*if…we could say ‘Look, this is where we’ve used it and it’s worked really well’, they’re more likely to pick it up*” (M13)) and the full range of strategies in each podcast topic (“*being able to make the connection with what the podcasts do to what we already do I think will be helpful in helping it be successfully implemented*.” (SP1)). Observing and discussing scenarios was suggested as an alternative to roleplays to better facilitate learning “*A detailed scenario… sometimes is a bit more engaging because you look at it and you pick it apart…rather than just the reciting back and forth*.” (SP22). One manager participant reflected on a previous experience of implementing an innovation, and suggested providing a package of materials for supervisors to lead reflective practice or discussion over PaRK-Lite, i.e., with reflective prompts. Finally, a preference for group or peer-to-peer learning was reported, as participants considered that this approach facilitates learning from trusted perspectives.

*It’s great to get that perspective from someone else and how they might implement it…they might have completely different ideas*.

(SP17)

*From a leadership position…they’ll say ‘But you don’t know how it’s going to work.’ Or ‘We don’t have the time.’ If it’s coming from a peer…they can share what they’ve learnt along the way to make it work*.

(M13)

### 3.13. Implementation Process Domain

The CFIR’s *Implementation Process Domain* construct captures constructs relating to the activities and strategies needed to implement PaRK-Lite.

### 3.14. Implementation Process Domain Facilitators

Planning: measuring engagement in a small-scale pilot. One participant expressed that, since “*community health only has a very limited amount of funding*” (M15), implementation plans should be on a small scale initially, using these evaluation outcomes to guide decision-making around scaling up. Identifying appropriate target client groups and referral pathways into the services that work with those client groups in advance was viewed as a facilitator for implementation: *“It will be a bit in the skill of us matching… once we know that, we’ve got all the systems in place to be able to refer through to that group of people.*” (SP14). There was also a suggestion that specific plans for delivering PaRK-Lite be made at the team level to best fit team-specific work processes and practices. Suggested outcomes to measure during implementation included: invitation rates, attendance rates, changes in risk level, parent confidence, and/or motivation to stay engaged with PaRK-Lite. “*Who’s coming through the door? What are they accessing? How long are they spending? Really interrogate that client pathway and different flows through.*” (M12).

Reflecting and evaluating: Protecting time for intervention deliverers and recipients to reflect and evaluate. Participants emphasised the importance of protecting time to share information and experiences relevant to evaluating PaRK-Lite’s implementation (“*it’s about being able to take stock and reflect*.” (SP9)) during team meetings or individual supervision, “*Workers are busy, they have to go to team meetings, so they’re all going to be there, you’ll have their attention, and I just think it really helps.*” (SP10). Likewise, participants emphasised protecting space during micro-coaching sessions for parents to share their experiences of engaging with it. As illustrated by M12:

*The end user voice…would be really important as we might be able to tailor it or pivot it, in a way that we think is most appropriate if it’s not hitting the mark for some cohorts but it is for others. Having all of that information is going to be really quite critical to the success of it*.

Engaging staff: Embedding implementation plans into existing practices. Service provider participants reported that sufficient time to both learn and integrate PaRK-Lite into their existing workflows, and providing quick and easy access to program materials and resources, would reduce the likelihood that PaRK-Lite would overwhelm their existing workload and encourage them to implement PaRK-Lite. This was echoed by manager participants, with M11 stating “*[having] the supports and the tools to do it easily so that there are no barriers…that sort of thing is really important*.”

Engaging staff: endorsing benefits. Participants reflected that embedding PaRK-Lite as a formal service offering would help staff understand and remember it as a complementary, rather than additional, piece of their workload: “*the best way to make this stuff work is to make people aware it’s there and help them understand how to use it, but then to embed it in their workflow so they don’t forget.*” (M15). Participants also discussed how team meetings may further facilitate building both internal and external motivation to participate in PaRK-Lite’s implementation. As described by M13, “*If you can bring it back to that value and that it’s improving the work that they’re doing and outcomes for the families, then that’s going to be a huge benefit*.”

Engaging recipients: Endorsing intended benefits and encouraging ongoing engagement. Participants described several strategies that could encourage parental uptake when introducing PaRK-Lite, including highlighting PaRK-Lite’s intended benefits for parents and children in general, and demonstrating how to access the podcasts, “*It highlights the importance if they’re sitting together doing it…I also think it then shows the commitment of the IPC staff member too.*”(M13). Participants reflected on experiences of less successful parenting interventions, where goals and timelines for intervention completion were ambiguous, “*I think what I’ve learnt is that sometimes being just a bit too ambiguous about things doesn’t get things done*” (SP19), and highlighted the importance of letting parents set their own timelines to encourage ongoing engagement.

### 3.15. Implementation Process Domain Barriers

Engaging recipients: lack of translated versions. Participants reported that PaRK-Lite only being available in English will likely present a barrier as “*We do have a lot of people who don’t have English as their first language. Or aren’t able to read English. I think some translated resources would be good*.” (M13)

Unclear outcomes and feedback. Participants discussed how insufficient or unclear outcome measures and feedback processes could mean “*People might not be clear of what the value of it is*” (M12). For example, existing ambiguities around documenting time spent implementing the digital components of PaRK-Lite was raised by M15 as a significant barrier to evaluating its value for larger-scale implementation, which was especially important given the little set-up funding available for digital initiatives: *“If it costs us money to implement [digital] solutions but we can’t stat the time, then we don’t get the benefits of being able to increase the capacity of our human resources because we can’t actually document [their] value*.”

Difficulty evaluating fidelity. Participants emphasised the importance of fidelity in evaluating outcomes (“*so that it is PaRK-Lite that we’re actually implementing*” (SP14)) and reported a wariness that the micro-coaching’s highly flexible delivery design could be a barrier to assessing fidelity.

### 3.16. Outer Setting Domain

The CFIR’s *Outer Setting Domain* construct captures constructs relating to the setting in which the Inner Setting exists, i.e., the community health service’s linkages with other health and community services and academic institutions. One manager participant spontaneously reflected on the partnership and connection between the community health service and the research institution, and expressed several barriers to engaging staff as co-designers that warrant consideration for future partnerships.

### 3.17. Outer Setting Domain Barriers

Partnerships and connection: Momentum and relevance. M24 expressed that remote working conditions (due to COVID-19) contributed to “*[losing] momentum*” and that the research’s intended end-goal and benefits for staff were not communicated with sufficient relevance to pique staff engagement. They acknowledged: *“you don’t have an end product, but that capacity to be able to brainstorm to see how they could be using [it]*” may facilitate staff involvement for future collaborations. They also expressed that the co-design research methods may not have been positioned as sufficiently relatable to staff’s everyday work, “*when you’re doing on the ground stuff…you’re not really thinking about research and co-designing…it’s just almost a foreign world.*”

### 3.18. Implementation Strategies

CFIR barriers identified by participants were matched with theory-informed implementation strategies using the publicly accessible CFIR–ERIC Matching tool. Table 2 describes CFIR barriers identified by participants, definitions of these barriers per the CFIR and specific to participants in this study, and a suggested ERIC implementation strategy or strategies to address each barrier.

## 4. Discussion

### 4.1. Main Findings

This research aimed to help bridge the research-to-practice gap between evidence-based, technology-assisted parenting interventions targeting family-level ACEs and services that are ideally placed to deliver these interventions. To the best of our knowledge, this is the first study to attempt addressing the parenting intervention research-to-practice gap by using the CFIR–ERIC tool to describe relevant barriers, facilitators and implementation strategies for embedding evidence-based innovations into services working with families of children experiencing adversity. Fourteen CFIR constructs were identified as facilitators. Eleven CFIR constructs were also identified as barriers, and by using the CFIR–ERIC tool, eleven strategies to mitigate these barriers were identified. The majority of strategies belonged to the ERIC clusters *Use evaluative and iterative strategies* (*n* = 4) and *Develop stakeholder interrelationships* (*n* = 3), suggesting the importance of ongoing assessment from multiple perspectives in implementing innovative interventions in these settings. The results of this study form the basis for an implementation plan to aid decision-making around adopting PaRK-Lite, and, if adopted, to improve the uptake of PaRK-Lite, with potential relevance to other similar interventions.

### 4.2. Facilitators

Individuals domain. Endorsing PaRK-Lite and fostering collaborative decision-making around delivery plans were reported as characteristics of high-level leaders that would facilitate implementation. Prior parenting intervention implementation studies have also found that organisation-level endorsement and expectations around use are linked to service providers’ sustained use of the parenting intervention [26]. An additional implementation strategy could thus include organisation-level endorsement of PaRK-Lite to staff prior to implementation. Inherent operational leadership skills were reported as facilitators for mid-level leaders (such as service managers and team leaders) to carry out their roles in PaRK-Lite’s implementation. Operational leaders carry out hands-on, day-to-day management of teams and organisations, and by virtue of this, are argued to be best placed for leadership duties such as defining organisational priorities [53]. While prior research has reported that leadership is critical to uptake of new parenting programs in the context of childhood adversity [54], no empirical research has been conducted on the role of operational leadership on implementation. The literature may, therefore, benefit from studies examining how different types of leadership are linked with implementation outcomes such as uptake.

Prior experience with parenting, family work and coaching was reported as important for facilitating service providers’ delivery of PaRK-Lite. This is consistent with past research, which found that confidence and self-efficacy in conducting parenting interventions is significantly associated with intervention delivery [55,56]. Openness and interest in innovative ways of working and ensuring staff feel the opportunity to deliver PaRK-Lite is their choice based on their perceived capacity were also reported as facilitators to delivering PaRK-Lite. Positive attitudes towards changes in practice have previously been linked with more favourable attitudes towards adopting innovations [55]. Consistent with the ERIC strategy of ‘*assessing for readiness*’, such attitudes may, therefore, represent an important aspect to assess when determining the degree of readiness to implement PaRK-Lite across teams within the community health service.

Digital literacy, internal motivation to engage, and awaiting services while on a waitlist were reported as characteristics that would likely facilitate parents’ engagement in PaRK-Lite. Research has indicated that podcast listeners tend to be tertiary-educated [57], suggesting they may appeal more to individuals with higher levels of education or digital literacy. However, spontaneously identified by participants in this study, demonstrating how to access the podcasts during service contacts may be a strategy to overcome potential digital literacy barriers and facilitate parents’ engagement. Receiving brief interventions while on a waitlist has previously been reported to increase consumers’ sense of agency over their wellbeing, and improvements in target clinical outcomes [58], hence implementing PaRK-Lite in waitlist spaces appears warranted.

Innovation domain. PaRK-Lite’s brief and modular format, its informal and entertaining use of sports commentary genre to deliver information, and its hybrid structure of a self-guided podcast and interactive micro-coaching components were reported as relative advantages that could facilitate adapting the delivery of PaRK-Lite to best meet parents’ preferences, including potentially including it as part of child-focused work. Adaptability has been identified in recent systematic reviews of parenting intervention implementation studies and evidence-based intervention studies as a key facilitator of implementation, or, conversely, a lack of program adaptability as a key barrier [25,59]. Thus, framing PaRK-Lite’s relative advantages as adaptable may be an additional strategy to encourage uptake by service providers.

Inner setting domain. PaRK-Lite’s intended purpose (enhancing engagement with parenting support), target recipients (parents of children aged 4–11) and outcomes (improving family wellbeing) was viewed by participants as being strongly aligned with the community health service’s overarching purpose and strategic objectives. This was viewed as a facilitator to its adoption, consistent with past research [25]. A good fit with the types of parenting support already offered, digital and practical resources available, and the work practices and processes already in place for individual client work was also viewed as a facilitator, supporting the potential value of using existing work practices as a design constraint for adapting intervention delivery formats [45]. Service providers’ perceived reward from noticing and celebrating parenting-related achievements during micro-coaching was also viewed as a facilitator to delivering PaRK-Lite. Past research has also identified client progress feedback as a positive reinforcement mechanism for practitioners implementing evidence-based practices in child welfare [26]. Given the high risk of burnout in child and family welfare workers [60], this rewarding aspect may represent an additional strategy to encourage uptake by service providers.

Regarding training, participants expressed a desire to understand PaRK-Lite’s origins, content, and delivery processes in full, as well as leveraging peer-to-peer learning among service providers. It has been proposed that the mutual trust and honesty fostered between peers with comparable status in an organisation supports learning by building motivation and proactivity [61]. Given the importance of quality training as a facilitator to implementation and a predictor of effective parenting program delivery [25,56,62], the role of peer-to-peer training warrants further investigation.

Implementation process domain. Participants identified clear ideas on implementation outcomes of interest that could be measured in a small-scale pilot, and inform decision-making around wider adoption and scale-up in the community health service. Protecting time during existing meeting spaces (such as supervision or team meetings) was highlighted as a potential facilitator of generating quantitative and qualitative data to support PaRK-Lite’s ongoing delivery and evaluation. Past research has also found that regular case conference and consultation was significantly predictive of more frequent program delivery during implementation [26], which may be due to the consolidation in learning and resulting confidence to deliver new programs. Strategies to engage service providers in implementation were characterised by the extent to which PaRK-Lite could be seamlessly integrated into existing service provision and work practices, whereas strategies to engage parents in receiving PaRK-Lite during implementation were characterised by building parents’ motivation. These findings inversely echo previous research, which found that the ineffective incorporation of new parenting programs into existing practices, and low client motivation to engage, are key barriers to the implementation of such programs [25].

### 4.3. Barriers

Innovation domain. Socioeconomic disadvantage and internet outage issues were reported as potential relative disadvantages and barriers to parents engaging with PaRK-Lite’s podcasts. While recent data indicate that Australian citizens who experience socioeconomic disadvantages (such as not having completed secondary schooling or living in public housing) are considered ‘highly excluded’ from digital services [63], families on low incomes are equally if not more likely to rely on mobile phones as a means of communication compared to other socio-economic positions [63,64]. PaRK-Lite’s podcasts being accessible via smartphones may, therefore, mitigate this. Additionally, we suggest conducting a local needs assessment as an appropriate ERIC strategy to collect and analyse data related to digital literacy and access barriers for parents living in the LGAs served by the community health service. Design quality, in the context of using sporting commentary as a genre to present evidence-based information, was also reported as a potential barrier to engaging families. We suggest that promoting adaptability by co-designing additional materials to present the evidence-based content may help promote adapting PaRK-Lite in a way that preserves fidelity. This ERIC strategy appears to be especially important given the importance of adaptability in facilitating implementation [25,59].

Inner setting domain. Longstanding full caseloads and waitlists were cited as existing barriers to integrating innovations like PaRK-Lite into work infrastructures. This finding represents a first step in implementing the suggested ERIC strategy to mitigate this barrier, namely, assessing for readiness and identifying barriers and facilitators*,* which we suggest is critical to continue on a team-by-team basis to ensure PaRK-Lite’s initial, small-scale implementation occurs in a team setting deemed ready. Allowing ample time to sensitively conduct conversations about parenting was viewed as a potential barrier to the micro-coaching’s compatibility with existing work practices. Micro-coaching is a novel construct proposed to enhance motivation and behavioural change in adult learning contexts [65], and, to the best of our knowledge, no existing intervention or study has implemented micro-coaching in the context of parenting interventions. For this reason, we suggest the ERIC strategy of conducting local consensus discussions and subsequently promoting adaptability will be critical to evaluating if and how conversations about parenting should extend beyond a micro-coaching session, and in what ways PaRK-Lite could be tailored to address this need where necessary. These strategies may be especially important, given poor integration of a program with existing caseloads and responsibilities has been identified as a key barrier to implementing parenting programs in real-world settings [25].

The relative priority of addressing social complexities and risk issues was highlighted by participants as a potential barrier to delivering PaRK-Lite. Prior research has also found that service providers’ perception of complex issues and family dynamics can be a barrier to delivering in parenting programs, although comparatively few parents identified such a barrier [66]. We therefore suggest parent stakeholders be involved with the ERIC strategy of conducting local consensus discussions to clarify if and how social complexities are a barrier to engaging with parenting work, and to subsequently clarify PaRK-Lite’s relative priority in these instances.

The training and learning time required to deliver PaRK-Lite was cited as a potential disincentive and barrier by staff, given the volume of training undertaken over the last few years. The risk of practitioner burnout increased during COVID-19 as practitioners endeavoured to maintain safeguarding families while swiftly pivoting longstanding work practices [60]. To avoid further exacerbating this risk, it is important that PaRK-Lite’s training does not feel onerous for service providers. We suggest working closely with staff to alter incentive structures and identify how PaRK-Lite training could incentivize practitioners as a strategy to facilitate this. For example, findings from this study suggest that minimal training time is one possible incentive. PaRK-Lite deliverers not having authority to procure digital resources for families if needed was cited as a potential barrier to resource availability. While the ERIC strategies suggested to overcome this included accessing new funding and changing physical infrastructure and equipment*,* we instead suggest that the previously mentioned ERIC strategy of conducting a local needs assessment to collect and analyse data related to digital access barriers for parents living in the LGAs served by the community health service is first needed to assess if these ERIC strategies are needed to mitigate this barrier.

Implementation process domain. Unclear plans for outcome measurement and communication were cited as potential barriers to evaluating PaRK-Lite’s small-scale implementation, and hence establishing its wider value for the community health service. We suggest developing a formal implementation blueprint, including appropriate performance and progress measures as an ERIC strategy to prevent this. Ambiguity around measuring fidelity and time spent implementing the digital components of PaRK-Lite were also cited as potential barriers to effectively reflect and evaluate PaRK-Lite’s efficacy. PaRK-Lite’s design intended to balance flexible delivery formats with fidelity to evidence-based content by pairing non-negotiable, evidence-based content (i.e., PaRK-Lite’s podcasts) with components that support delivering content in a highly contextual manner (i.e., PaRK-Lite’s micro-coaching) [67]. In addition to quantitative engagement measures tracking fidelity to the podcasts, we suggest that the ERIC strategy develop and implement tools for quality monitoring to define then measure the micro-coaching’s intended processes and outcomes, such as parents’ perceived empathy and rapport with their service provider. Developing and using tools to ensure time spent implementing digital aspects of PaRK-Lite can be documented is also needed, which was reported by one manager participant as an identified challenge to address.

English-only resources were viewed as a barrier to engaging parents from culturally and linguistically diverse backgrounds during implementation. However, prior research with a culturally diverse sample of parents in Australia found that parenting program content and materials were perceived as culturally appropriate, and key barriers to their engagement were more related to practical aspects to accessing such programs [68]. We suggest involving consumers to firstly identify which aspects of PaRK-Lite might interfere with their engagement, then guide the development of culturally appropriate, translated resources that are likely to attract and engage people from diverse backgrounds.

Outer setting domain. Remote working conditions and ambiguity around PaRK-Lite’s research process and intended end-goal and benefits for staff were identified by one manager participant as barriers to establishing strong connections between the community health service and the academic institution. Co-design research methods embrace ambiguity through iterative prototyping, which can often be at odds with the evidence-driven approach familiar to health contexts [69,70]. We therefore suggest focusing on building a coalition as a strategy to mitigate this in future, by first exploring service providers’ motivation or stakes in the potential co-designed outcomes to generate a shared intended end-goal and benefit of the co-design process. This shared motivation between service providers and researchers from the academic institution can anchor the partnership and potentially support cultivating a stronger connection.

Barriers specific to technology-assisted components. The technology-assisted components of PaRK-Lite’s hybrid design (i.e., podcasts) were novel to the community health service, hence barriers specific to these components provide novel insights into implementing technology-assisted components that may be of relevance to other community health services considering innovating service provision with technology. This study found that barriers to implementing the podcasts were characterised by parents facing potential difficulties with digital literacy and access to smartphones required to listen to them, and service providers’ potential difficulties in procuring smartphones for families if needed. The ERIC strategy of conducting a local needs assessment emerged for these barriers only, underscoring the importance of collecting and analysing data related to users’ perceived advantage and available resources for engaging with novel, technology-assisted components.

PaRK-Lite’s first design iteration involved firstly working with service providers to understand how to facilitate implementation of technology-assisted components that are novel to their service, then developing prototype technology-assisted components and delivering them to parents on a small scale with a view to collecting and analysing feedback data to inform the next design iteration. Findings from this study suggest that conducting a local needs assessment of parents’ perceived advantage and resources for using technology-assisted components may complement collection of feedback data. Conducting a local needs assessment may, thus, represent both a helpful initial design and a pre-implementation strategy for creating an innovative, meaningful, but feasible prototype for wider-scale implementation and evaluation.

### 4.4. Practical Implications

Findings from this study will be shared with the community health service, and ongoing discussions with a dedicated stakeholder group (comprised of service managers and researchers) will be held to continue operationalising implementation strategies. The Action, Actor, Context, Target, Time (AACTT) framework [71] will be used to support operationalisation. In doing so, these findings will support the small-scale implementation and evaluation of PaRK-Lite to, in turn, support decision-making around wider implementation and scale-up at the community health service. Additionally, findings from the current study are highly consistent with past research into facilitators and barriers to implementing evidence-based interventions, hence, facilitators and barriers identified in this study are likely to be of practical relevance to other settings that serve families of primary-school-aged children seeking to successfully implement innovative parenting support.

### 4.5. Strengths and Limitations

Strengths. Qualitative data are limited in their generalisability to other contexts. However, the use of the CFIR–ERIC taxonomy in this study facilitates a comparison with barriers and facilitators in different but related contexts, contributing to systematic appraisals of implementation factors in the field of implementation science [72]. Managers from each relevant service participated in this study, enhancing the representation of barriers and facilitators relevant to this group of implementation participants.

Limitations. Firstly, the number of service provider participants from each service was small relative to the number of staff in that service (i.e., 5/28). Understanding barriers and facilitators to implementation from a wider range of staff may have enriched our descriptions of identified barriers and enablers, and the selection of implementation strategies to address barriers. Secondly, the evidence base underpinning the CFIR–ERIC tool is somewhat heterogeneous in terms of the expert endorsement of strategies to address barriers, meaning other clinical and academic implementation experts may differ in their endorsement of strategies described in the current study. We aim to overcome this with our intention to discuss and operationalise ERIC strategies identified in the current study with the community health service staff to ensure they are adequately contextualised and compatible aims.

### 4.6. Future Research

Strategies for overcoming the majority of CFIR barriers identified in this study belonged to the ERIC clusters *Using evaluative and iterative strategies* and *Developing stakeholder interrelationships* (see Table 2). These clusters are consistent with co-design principles and the ‘Double Diamond’ framework for innovation that guided PaRK-Lite’s design, namely, collaborating and iterating [73]. Thus, future research endeavouring to reduce research-to-practice gaps using the ‘Double Diamond’ framework for innovation are recommended to embed evaluative and iterative strategies and focus on developing stakeholder interrelationships. Ongoing collaboration with stakeholders should also involve process evaluations of the proposed ERIC implementation strategies to understand both if and why some strategies support implementation while others fail to change practice. Such evaluations play an especially critical role in small-scale implementation projects through offering insight into the feasibility and applicability of such strategies for scale-up, and providing an opportunity to revise implementation plans as needed [74].

## 5. Conclusions

Using the CFIR–ERIC approach facilitated the identification of relevant and concise pre-implementation strategies for addressing potential barriers to implementing a novel, co-designed parenting intervention for parents of children with ACEs. Facilitators identified by participants in the Innovation and Inner Setting domains supported the value of co-design as an initial phase in bridging research-to-practice gaps, as it facilitates designing innovations that fit with and enhance existing practices, which, in turn, facilitate implementation. Findings were largely consistent with prior research, suggesting that applying these strategies will support successful implementation outcomes such as uptake and engagement with PaRK-Lite, and may be used by other healthcare settings aiming to innovate services with technology-assisted parenting interventions to improve child mental health.

## Figures and Tables

**Table 1:** Participant demographics (*n* = 14).

Characteristics	*n*	%
Age (years)		
25–34	1	7
35–44	8	57
45–54	3	21
55–64	2	14
Experience (number of years in role)		
0–5	1	7
6–10 years	2	14
11–15 years	3	21
16–20 years	4	28
21–25 years	0	0
26–30	2	14
31–35	2	14
Gender		
Male	2	14
Female	12	86
Discipline		
Social Work	7	50
Occupational Therapy	1	7
Psychology	1	7
Management + Clinical (Allied Health)	5	36
Management + Executive Leadership	1	7

**Table 2:** CFIR, study-specific barriers, and ERIC implementation strategies suggested by the CFIR–ERIC matching tool.

CFIR Construct Characteristic: CFIR Barrier Definition	Study-Specific Definition	ERIC Strategy Cluster	ERIC Strategy: *Definition*	Study-Specific Definition
Innovation domain Relative advantage: *Stakeholders do not see the advantage of implementing the innovation compared to an alternative solution or keeping things the same*	Digital literacy and access are ongoing barriers for parents in the Wyndham LGA	Use evaluative and iterative strategies	Conduct local needs assessment: *Collect and analyse data related to the need for the innovation*	Collect and analyse data related to digital literacy and access barriers for parents living in the LGAs served by the community health service
Innovation domain Design quality and packaging: *Stakeholders believe the innovation is poor quality based on the way it is bundled, presented, and/or assembled*	The design of the podcast using sporting commentary may be a poor match for families who are not interested in sport, or who do not understand sporting metaphors.	Adapt and tailor to context	Promote adaptability: *Identify the ways a clinical innovation can be tailored to meet local needs and clarify which elements of the innovation must be maintained to preserve fidelity*	Co-design additional materials with parents to present the evidence-based content in different ways
Inner setting domain Work infrastructure: *The social architecture, age, maturity, and size of an organization hinders implementation*	Existing challenges to service functioning: full caseloads and long waitlists, and leadership team and practitioner team’s scope of practice still being developed	Use evaluative and iterative strategies	Assess for readiness and identify barriers and facilitators: *Assess various aspects of an organization to determine its degree of readiness to implement, barriers that may impede implementation, and strengths that can be used in the implementation effort*	Assess readiness on a team-by-team basis
Inner setting domain Compatibility: *The innovation does not fit well with existing workflows nor with the meaning and values attached to the innovation, nor does it align well with stakeholders’ own needs and/or it heightens risk for stakeholders*	Time needed for sensitive parenting work: parenting discussions can trigger larger conversations, which may not feasibly fit into 15-min micro-coaching within existing service contact	Develop stakeholder interrelationships	Conduct local consensus discussions: *Include local providers and other stakeholders in discussions that address whether the chosen problem is important and whether the clinical innovation to address it is appropriate*	Discuss with stakeholders, including parents, if and how conversations about parenting should extend beyond a micro-coaching session
		Adapt and tailor to context	Promote adaptability: *Identify the ways a clinical innovation can be tailored to meet local needs and clarify which elements of the innovation must be maintained to preserve fidelity*	Assess in what ways PaRK-Lite could be tailored to address extending micro-coaching sessions if necessary
Inner setting domain Relative priority: *Stakeholders perceive that implementation of the innovation takes a backseat to other initiatives or activities*	Prioritising complex social needs: Social complexities can interfere with parents’ readiness to engage with parenting support and service providers’ willingness to provide it	Develop stakeholder interrelationships	Conduct local consensus discussions: *Include local providers and other stakeholders in discussions that address whether the chosen problem is important and whether the clinical innovation to address it is appropriate*	Clarify if and how social complexities are a barrier to parents’ engagement in discussions about their parenting
Inner setting domain Incentives and rewards: *There are no tangible (*e.g.,* goal-sharing awards, performance reviews, promotions, salary raises) or less tangible (*e.g.,* increased stature or respect) incentives in place for implementing the innovation*	Training fatigue: Time needed for training and delivering PaRK-Lite is a potential disincentive due to large volume of training taken in recent years, and consistently full caseloads.	Utilise financial strategies	Alter incentive/allowance structures: *Work to incentivize the adoption and implementation of the clinical innovation*	Identify how practitioners could be incentivised to receive training and deliver PaRK-Lite
Inner setting domain Available resources: *Resources (*e.g.,* money, physical space, dedicated time) are insufficient to support implementation of the innovation*	Timeliness of procuring digital resources: requires managers’ approval and oversight.	Use evaluative and iterative strategies	Conduct local needs assessment: *Collect and analyse data related to the need for the innovation*	Collect and analyse data related to digital literacy and access barriers for parents
Implementation process domain Planning: *A scheme or sequence of tasks necessary to implement the intervention has not been developed or the quality is poor*	Unclear outcome measures and feedback processes: Ambiguity around documenting time spent implementing digital aspects of PaRK-Lite may be a barrier to feeding back quantitative measures and progress and a barrier to perceived value	Use evaluative and iterative strategies	Develop a formal implementation blueprint: *Develop a formal implementation blueprint that includes all goals and strategies. The blueprint should include the following: (1) aim/purpose of the implementation; (2) scope of the change (*e.g.,* what organizational units are affected); (3) timeframe and milestones; and (4) appropriate performance/progress measures. Use and update this plan to guide the implementation effort over time*	Include appropriate performance and progress measures
Implementation process domain Reflecting and evaluating: *There is little or no quantitative and qualitative feedback about the progress and quality of implementation nor regular personal and team debriefing about progress and experience*	Difficulty evaluating fidelity: Micro-coaching’s highly flexible delivery design may be a barrier to assessing qualitative measures such as fidelity	Use evaluative and iterative strategies	Develop and implement tools for quality monitoring: *Develop, test, and introduce into quality-monitoring systems the right input—the appropriate language, protocols, algorithms, standards, and measures (of processes, patient/consumer outcomes, and implementation outcomes) that are often specific to the innovation being implemented*	Define then measure the intended processes and outcomes of PaRK-Lite’s micro-coaching, such as parents’ perceived empathy and rapport with their service provider
Implementation process domain Engaging: *Multi-faceted strategies to attract and involve key stakeholders in implementing or using the innovation (*e.g.,* through social marketing, education, role modeling, training) are ineffective or non-existent.* *Multi-faceted strategies to attract and involve patients/customers in implementing or using the innovation (*e.g.,* through social marketing, education, role modeling, training) are ineffective or non-existent*	Lack of translated versions: PaRK-Lite only being available in English will not attract or include parents from culturally and linguistically diverse backgrounds	Engage consumers	Involve patients/consumers and family members: *Engage or include patients/consumers and families in the implementation effort*	Involve parents to identify which of PaRK-Lite’s content and delivery components might interfere with their engagement, then develop culturally appropriate, translated resources
Outer setting domain Partnerships and connections: *The organization is not well networked with external organizations*	Remote or work-from-home conditions detract from momentum, intended end-goal and benefits of action research were not communicated with sufficient relevance to staff	Develop stakeholder interrelationships	Build a coalition: *Recruit and cultivate relationships with partners in the implementation effort*	Explore service providers’ motivation or stake in potential outcomes to generate an intended end-goal and benefit of the co-design process

## Data Availability

The original contributions presented in this study are included in the article/supplementary material. Further inquiries can be directed to the corresponding author(s).

## Supplementary Materials

The following supporting information can be downloaded at: https://www.mdpi.com/article/10.3390/ijerph21121599/s1; Interview Schedules.
