# Supplementary material for: Developing Pre-Implementation Strategies for a Co-Designed, Technology-Assisted Parenting Intervention Using the Consolidated Framework for Implementation Research (CFIR) and Expert Recommendations for Implementing Change (ERIC) Approach
Source: Int J Environ Res Public Health. 2024 Nov 30;21(12):1599. doi: 10.3390/ijerph21121599 (PMC11675243; doi:10.3390/ijerph21121599)
Supplement: Supplementary file 1 [file ijerph-21-01599-s001.zip › ijerph-3190004-supplementary.pdf]

Supplementary Material 1

Interview Schedules

Service Provider Interview Schedule

Research Aims:

1) To explore the barriers and facilitators to implementing PaRK-Lite at IPC Health
2) To devise a set of actionable strategies to support implementation of PaRK-Lite at IPC Health

Research Questions:

- What are the potential barriers and facilitators to implementation from a **service provider's** perspective?

Checklist / script for start of interview

- Thank participant(s) for attending
- Introduce self
    - Leading this project which is my PhD project. Just me involved, so have worked closely with SPs and now parents throughout the co-design
    - Also completed Psychology training, interest in trauma-informed family work
- Remind participants of purpose of interview
    - The purpose of this feedback chat is first and foremost to hear your **feedback** on the co-designed parenting program you learnt about in the video. **When** did you last watch the video? A reminder that the podcasts **aim** to deliver the evidence-based parenting information and strategies, and the purpose of the micro-coaching is to give parents a place to pause, reflect and connect with what the podcasts are saying. Your role in the micro-coaching is simply to facilitate that space.
    - What we're really hoping to understand at this stage too is what kinds of things you think will **help or hinder implementing** PaRK-Lite's podcasts and micro-coaching into your service provision.
    - By deeply understanding this, we're hoping to make some **refinements to the final product** where possible and come up with a range of strategies that will help set implementing PaRK-Lite up for success.
    - It might feel awkward providing feedback that isn't supportive, but this stage is really about learning about how we can make further improvements. As such, I would really like you to be **honest with your feedback**, and encourage you to speak as freely as you can - **I will not take any feedback personally**. Your feedback is incredibly valuable to the process of innovating parenting support.
- Explain that we have some set questions to ask, but also that we would like to hear any open feedback too.
- Explain that overall interview will take ~45mins, but it's OK if it's shorter or longer, we will be guided by how much feedback they have. Check in with the participant about any time constraints that they may have.
- Explain that they can change their mind and stop at any time – just let us know.

- Remind about recording. Explain:
  - Recording so that we can transcribe what is said and have an accurate record for our research.
  - The recording will be password protected and stored securely on Monash University servers
  - Only accessible by the research team
  - Stored separately to identifiable information about you, (e.g. your contact details)
  - ASK if they have any questions about this.
- Once confident they understand and consent, explain that you will start recording and then will ask their consent again, for the recording.
- START RECORDING ***Repeat consent questions for recording.*** "Just so I have it on the recording, I'm going to ask again. Based on what I've just explained, are you still happy to take part in this interview, including the recording? Great, thank you. Let's get started…"

**CFIR Domain/Sub-constructs**<br><br>Priority 1 Questions<br><br>Priority 2 Questions	**Question**
**N/A**<br><br>Feedback to be mapped onto a relevant CFIR construct.	**Open-ended opening question:**<br><br>• To start with, it would be great to hear about your overall impressions of PaRK-Lite. What is the main thing you'd like to feedback about PaRK-Lite? **[PROBE]**<br>• What are some of the key considerations that come to mind when it comes to implementing an innovation like PaRK-Lite? By implementing, I mean taking up new practices as part of your usual or routine practices with the hope of improving the overall quality and effectiveness of the service you provide. **[PROBE]**<br>• What can service providers do to support parent's engagement with PaRK-Lite? By engagement, I mean choosing to start PaRK-Lite and stick with it until completion. **[PROBE]**

**Intervention characteristics**<br>**(CFIR 2.0 'Innovation Domain')**<br>• Relative advantage<br>• Adaptability<br><br>• Complexity<br>• Evidence strength and quality<br>• Design quality and packaging<br>• Costs	• **Relative advantage.**<br>○ How does PaRK-Lite's podcasts and micro-coaching **compare** to other parenting support options that may have been considered, or that you know about at IPC Health? **[PROBE]**<br>• **Adaptability.**<br>○ What kinds of changes or **alterations** do you think will need to be made so that PaRK-Lite will work effectively in your service? **[PROBE]**<br><br>• **Complexity.**<br>○ If you imagine implementing PaRK-Lite into your usual service provision, how **complicated** do you think it would be?<br>• **Evidence strength and quality**<br>○ What kinds of supporting evidence or proof do you think is needed about the effectiveness of PaRK-Lite, to get staff on board with implementing it along with other services?<br>• **Design quality and packaging**<br>○ What is your perception of the quality of how the podcasts and micro-coaching material are presented and packaged for staff?<br>○ How might these materials affect implementation in your service?<br>• **Costs**<br>○ What costs do you imagine could be incurred to implement PaRK-Lite?
**Inner setting**<br>• **Implementation climate**<br>○ Tension for Change<br>Relative Priority<br>○ Compatibility<br>Learning Climate<br>○ Organisational Incentives and Rewards<br>○ Goals and Feedback	• **Implementation climate.**<br>○ What do you think the general level of **receptiveness** in your service would be to implementing PaRK-Lite? **[PROBE]**<br>**Tension for Change**<br>○ How essential is PaRK-Lite to meet the needs of parents at IPC Health?<br>○ How do people feel about current programs or practices that are available for parents of primary-school aged children?<br>• **Relative Priority**<br>○ What kinds of things appear to have the highest priority for you and IPC Health?<br>○ To what extent might implementing PL take a backseat to other high-priority initiatives going on now?<br>• **Compatibility**<br>○ How well does it seem to **fit** with existing work processes and practices in the service you offer? **[PROBE]**<br>**(Follow up Q) Learning Climate**<br>○ To what extent do you feel like you can try new things to improve or build on your current work practice?<br>• **Organisational Incentives and Rewards**<br>○ What kinds of **incentives** are there to help ensure that implementing PL is successful? **[PROBE]**<br><br>• **Goals and Feedback**<br>○ How does implementing PL align with other organisational goals you currently have?<br>○ To what extent are goals set for other programs and initiatives that have been implemented, and to what extent are they monitored?

• **Readiness for implementation**<br>○ Leadership engagement (CFIR 2.0 High & Mid level leaders roles and characteristics)<br>○ Available resources<br>○ Access to knowledge and information.	• **Leadership engagement.**<br>○ What kind of support or actions would you expect from team leaders/managers at IPC Health to help make the PaRK-Lite implementation successful? **[PROBE:** What is needed? What is available?**]**<br>• **Available resources.**<br>○ Do you feel your team has enough resources to implement PaRK-Lite? Which resources are sufficient and which are currently insufficient? **[PROBE]**<br>• **Access to knowledge and information.**<br>○ What kind of training would you prefer to receive?<br>○ Do you think the information and materials for PaRK-Lite will be easy enough to access via Teams?
**Characteristics of individuals**<br><br>**(CFIR 2.0 'Individuals Domain, Characteristics')**<br><br>Self-efficacy	• **Self-efficacy.**<br>○ How confident do you feel you could deliver PaRK-Lite along with your usual services? **[PROBE]**
**N/A**<br><br>Feedback to be mapped onto a relevant CFIR construct.	**Open-ended closing question:**<br><br>• Is there anything else you'd like to feedback PaRK-Lite, or potential barriers and enablers to implementation?
**Conclusion**	• Conclude, summarise key insights<br>• Remind SP that if conversation has brought up anything stressful that they can contact EAP (per Information Sheet)<br>• Member checking transcripts- would you like to read over the transcript of our conversation and send back any corrections or clarifications or additions?<br>• Thank for time

Manager Interview Schedule

Research Aims:

3) To explore the barriers and facilitators to implementing PaRK-Lite at IPC Health
4) To devise a set of actionable strategies to support implementation of PaRK-Lite at IPC Health

Research Questions:

- What are the potential barriers and facilitators to implementation from a **manager's** perspective?

Checklist / script for start of interview

- Thank participant(s) for attending

- Introduce self
  - Leading this project which is my PhD project. Just me involved, so have worked closely with SPs and now parents throughout the co-design
  - Also completed Psychology training, interest in trauma-informed family work
- Remind participants of purpose of interview
  - The purpose of this feedback chat is first and foremost to hear your feedback on the co-designed parenting program you learnt about in the video.
  - What we're really hoping to understand at this stage too is what kinds of things you think will help or hinder implementing PaRK-Lite's podcasts and micro-coaching into your service provision.
  - By deeply understanding this, we're hoping to make some refinements to the final product where possible and come up with a range of strategies that will help set implementing PaRK-Lite up for success.
  - It might feel awkward providing feedback that isn't supportive, but this stage is really about learning about how we can make further improvements. As such, I would really like you to be **honest with your feedback**, and encourage you to speak as freely as you can - **I will not take any feedback personally**. Your feedback is incredibly valuable to the process of innovating parenting support.
- Explain that we have some set questions to ask, but also that we would like to hear any open feedback too.
- Explain that overall interview will take ~45mins, but it's OK if it's shorter or longer, we will be guided by how much feedback they have. Check in with the participant about any time constraints that they may have.
- Explain that they can change their mind and stop at any time – just let us know.
- Remind about recording. Explain:
  - Recording so that we can transcribe what is said and have an accurate record for our research.
  - The recording will be password protected and stored securely on Monash University servers
  - Only accessible by the research team
  - Stored separately to identifiable information about you, (e.g. your contact details)
  - ASK if they have any questions about this.
- Once confident they understand and consent, explain that you will start recording and then will ask their consent again, for the recording.
- START RECORDING ***Repeat consent questions for recording.*** "Just so I have it on the recording, I'm going to ask again. Based on what I've just explained, are you still happy to take part in this interview, including the recording? Great, thank you. Let's get started…"

**CFIR Domain/Sub-constructs**<br><br>Priority 1 Questions	**Question**

<table>
<tr><td>Priority 2 Questions</td><td></td></tr>
<tr><td>N/A
Feedback to be mapped onto a relevant CFIR construct.</td><td>Open-ended <u>opening</u> question:
To start with, it would be great to hear about your overall impressions of PaRK-Lite. What is the main thing you'd like to feedback about PaRK-Lite? [PROBE]
What are some of the key considerations that come to mind when it comes to implementing an innovation like PaRK-Lite? By implementing, I mean taking up new practices as part of your usual or routine practices with the hope of improving the overall quality and effectiveness of the service you provide. [PROBE]
What can IPC Health do to support parent's engagement with PaRK-Lite? By engagement, I mean choosing to start PaRK-Lite and stick with it until completion. [PROBE]</td></tr>
<tr><td>Intervention characteristics

Intervention characteristics
(CFIR 2.0 'Innovation Domain')
Relative advantage
Adaptability
Complexity
Evidence strength and quality
Design quality and packaging
Costs</td><td>Relative advantage.
How does PaRK-Lite compare to other parenting support options that may have been considered, or that you know about at IPC Health? [PROBE]
Adaptability.
What kinds of changes or alterations do you think will need to be made so that PaRK-Lite will work effectively in your service? [PROBE]
Complexity.
If you imagine implementing PaRK-Lite into your usual service provision, how complicated do you think it would be?
Evidence strength and quality
What kinds of supporting evidence or proof do you think is needed about the effectiveness of PaRK-Lite, to get staff on board with implementing it along with other services?
Design quality and packaging
What is your perception of the quality of how the podcasts and micro-coaching material are presented and packaged for staff?
How might these materials affect implementation in your service?
Costs
What costs do you imagine could be incurred to implement PaRK-Lite?</td></tr>
</table>

<table>
<tr><td>Readiness for implementation
Leadership engagement (CFIR 2.0 High & Mid level leaders roles and characteristics)
Available resources
Access to knowledge and information.</td><td>Available resources.
Do you feel your team has enough resources to implement PaRK-Lite? Which resources are sufficient and which are currently insufficient? [PROBE]
Leadership engagement.
What kind of support or actions is expected of team leaders/managers at IPC Health to help make the PaRK-Lite implementation successful? [PROBE: what is needed? What is available?]
Access to knowledge and information.
What kind of training would you prefer to receive?
Do you think the information and materials for PaRK-Lite will be easy enough to access via Teams?</td></tr>
<tr><td>Inner setting
Implementation climate
Tension for Change
Compatibility
Learning climate</td><td>Implementation climate.
What do you think the general level of receptiveness in your service would be to implementing PaRK-Lite? [PROBE]
(Follow up Q) Tension for Change
How essential is PaRK-Lite to meet the needs of parents at IPC Health?
How do people feel about current programs or practices that are available for parents of primary-school aged children?
Compatibility
How well does it seem to fit with existing work processes and practices in the service you offer? [PROBE]
(Follow up Q) Learning Climate
To what extent do you feel like you can try new things to improve or build on your current work practice as a manager?</td></tr>
<tr><td>Individual characteristics:

(CFIR 2.0 ‘Individuals Domain, Characteristics’)

Self-efficacy</td><td>Self-efficacy
How confident do you feel you could support staff to deliver PaRK-Lite, along with your existing management workload or duties?</td></tr>
<tr><td>N/A

Feedback to be mapped onto a relevant CFIR construct.</td><td>Open-ended <u>closing</u> question (leave 15 mins for)
Is there anything else you’d like to feedback PaRK-Lite, or potential barriers and enablers to implementation?</td></tr>
</table>

**Conclusion**	• Conclude, summarise key insights<br>• Remind SP that if conversation has brought up anything stressful that they can contact EAP (per Information Sheet)<br>• Member checking transcripts- would you like to read over the transcript of our conversation and send back any corrections or clarifications?<br>• Thank for time

Supplementary Material 2

Consolidated criteria for reporting qualitative studies (COREQ): 32-item checklist

**No. Item**	**Guide questions/description**	**Reported on Page #**
**Domain 1: Research team and reflexivity**		
Personal Characteristics		
1. Inter viewer/facilitator	Which author/s conducted the inter view or focus group?	8
2. Credentials	What were the researcher's credentials? E.g. PhD, MD	8
3. Occupation	What was their occupation at the time of the study?	8
4. Gender	Was the researcher male or female?	8
5. Experience and training	What experience or training did the researcher have?	8
Relationship with participants		
6. Relationship established	Was a relationship established prior to study commencement?	8

7. Participant knowledge of the interviewer	What did the participants know about the researcher? e.g. personal goals, reasons for doing the research	8-9
8. Interviewer characteristics	What characteristics were reported about the inter viewer/facilitator? e.g. Bias, assumptions, reasons and interests in the research topic	8

**Domain 2: study design**		
Theoretical framework		
9. Methodological orientation and Theory	What methodological orientation was stated to underpin the study? e.g. grounded theory, discourse analysis, ethnography, phenomenology, content analysis	9
Participant selection		
10. Sampling	How were participants selected? e.g. purposive, convenience, consecutive, snowball	7
11. Method of approach	How were participants approached? e.g. face-to-face, telephone, mail, email	7-8
12. Sample size	How many participants were in the study?	6
13. Non-participation	How many people refused to participate or dropped out? Reasons?	7
Setting		
14. Setting of data collection	Where was the data collected? e.g. home, clinic, workplace	9
15. Presence of non-participants	Was anyone else present besides the participants and researchers?	9
16. Description of sample	What are the important characteristics of the sample? e.g. demographic data, date	7
Data collection		
17. Interview guide	Were questions, prompts, guides provided by the authors? Was it pilot tested?	8
18. Repeat interviews	Were repeat inter views carried out? If yes, how many?	9
19. Audio/visual recording	Did the research use audio or visual recording to collect the data?	9
20. Field notes	Were field notes made during and/or after the inter view or focus group?	9
21. Duration	What was the duration of the inter views or focus group?	9
22. Data saturation	Was data saturation discussed?	N/A
23. Transcripts returned	Were transcripts returned to participants for comment and/or correction?	9
**Domain 3: analysis and findings**		
Data analysis		
24. Number of data coders	How many data coders coded the data?	9
25. Description of the coding tree	Did authors provide a description of the coding tree?	N/A
26. Derivation of themes	Were themes identified in advance or derived from the data?	9

27. Software	What software, if applicable, was used to manage the data?	9
28. Participant checking	Did participants provide feedback on the findings?	9
Reporting		
29. Quotations presented	Were participant quotations presented to illustrate the themes/findings? Was each quotation identified? e.g. participant number	12-22
30. Data and findings consistent	Was there consistency between the data presented and the findings?	12-22
31. Clarity of major themes	Were major themes clearly presented in the findings?	11
32. Clarity of minor themes	Is there a description of diverse cases or discussion of minor themes?	N/A

Supplementary Material 3

CFIR Facilitator Results and Exemplar Quotes

CFIR Domain	**CFIR Construct**<br>Study-specific facilitator theme	Exemplar quotes
Individuals	**High-level leaders**<br>Organisational endorsement	"The message coming top down is really important to encourage workers that it is worthwhile…that it's supported and you're supported to do it." (SP1)<br><br>"I was really encouraged in a positive way…that [management] want to think about new ways of working with families or how they can improve service delivery." (SP9)
	**High-level leaders**<br>Collaborative decision-making	"Being clear about what the team is trying to achieve and working with them on what they need to do to achieve those objectives. (M15)<br><br>"I would be hoping that…staff would own it and, kind of, drive it as well." (M24)
	**Mid-level leaders**<br>Team-level endorsement	"The best operational leadership comes from someone who is part of the team" (M15)<br><br>"I would be really leaning on the team leaders …They would be really the people who are overseeing the clinicians using this tool" (M11)
	**Mid-level leaders**<br>Operational support	"Supported …on an organisational level to put the time aside." (SP9).<br><br>"There needs to be that management-driven level of support." (SP1)
	**Deliverers**<br>Prior experience	"You want them to understand about good family-centred practice and good parenting principles." (M11).<br><br>"The skill level and experience that you need in order to effectively micro-coach, I

		think, is really important to consider." (SP19)
	**Deliverers**<br>Openness to innovation	"If there's something new then I'm always willing to try it, and because I'm always looking for the best outcome for the client." (SP22).<br><br>"Particularly people who have chosen to work at the Hub, [they] want to do things in a different way. So, I think that would make them more open to adopting an innovation." (M11)
Innovation	**Relative advantage**<br>Brevity	"I think it's better because of those short, sharp sessions. I think more people - and it's the age that we live in too- people lose attention really quickly." (M13)<br><br>"I don't really have a whole lot of short resources that are easy to deliver to parents." (SP17).
	**Relative advantage**<br>Hybrid structure	"Building a bridge between that face-to-face service… to something that a family or a parent can access in their own time…that's something that I hadn't really seen before… it helps to integrate people's learning…applying theory to practice." (SP9)<br><br>"The flexibility of working around what's best for the client, which you don't get with other programs" and "boost[ing] their confidence and self-esteem, that they can do it on their own." (SP1)<br><br>"They can rewind and revisit with a bit more space in their own time versus being in a session and trying to be pushed along." (SP14)<br><br>"Getting a more targeted and successful outcome [by] being authentic and client-focused." (SP14).
	**Innovation source**<br>Co-designed	"You've certainly looked at all of the research … you've done all of the work as well as understanding the context." (M13)

		"You've gathered information and consultation with other health professionals who are working on the grassroots…that's really important and valid." (SP16)
	**Adaptability**<br>Universal relevance	"When you look at family units trying to live together, those topics are often what come up." (M11)<br><br>"Those three topics that were mentioned are literally mainly the reasons for referral" (SP19)
	**Adaptability**<br>Modular structure	"The length of the podcast [and] length of the micro-coaching sessions could adjust up or down. The prompting questions in the templates could be altered as we [staff] learn" (M15)<br><br>"My experience with clients is often… checking in and then realising oh, we could be doing it in a bit of a different way." (SP19)
Inner setting	**Mission alignment**<br>Aligned with strategic objectives	"[It's] consistent with where the organisation is heading and its overall strategic objectives." (M12)<br><br>"The overarching goal is the hopes for my child… you want that as the guiding journey or roadmap for why you're participating in something. I think we do that with all of our services." (SP14)
	**Compatibility**<br>Fits with existing support options	"You can implement PaRK-Lite…then if PaRK-Lite doesn't work, then you have another resource, and so on…until you meet the desired outcome... it's great to have a diverse range of resources you can use to support families." (SP17)<br><br>"Micro-coaching can be inserted into that hour long consultation…so I think that it's very adaptable to the workplace. With the podcast…they're nice and short enough that the person working with that family could sit with them, play the podcast, and then do the micro-coaching in real time." (M13)

	**Incentives and rewards**<br>Celebrating achievements is rewarding	"It [micro-coaching] still keeps that activity happening. You're still achieving things…I like to achieve things and I like to see my clients achieve things." (SP22).<br><br>"Not even just from your own client, but from your colleagues…we're making a difference…I guess that shared reward." (SP8)
	**Available resources**<br>Sufficient digital and practical resources to implement on a small scale	"Most people…even from different socio-economic backgrounds…usually have access to a smartphone…that in itself is obviously a good thing when it comes to reducing barriers." (SP19)<br><br>"[The practical resource] was great because then that really saves us a lot of time of having to think about what we're going to ask" (SP22).<br><br>"I think we have a lot of the resources…[but] It's not something that we've done before in Family Services. So I think it will be one of those trial and errors on whether we've got the resources or not." (SP1)
Implementation process	**Planning**<br>Plan for measuring engagement in small scale pilot	"Minimised effort and investment until you have evaluated outcomes and then you scale up as you build confidence that you're going to achieve what you want to achieve… rather than spend big buckets of money on something that hasn't been proven…community health only has a very limited amount of funding." (M15)<br><br>"It will be a bit in the skill of us matching… once we know that, we've got all the systems in place to be able to refer through to that group of people." (SP14)<br><br>"Who's coming through the door? What are they accessing? How long are they spending? Really interrogate that client pathway and different flows through." (M12)

	**Engaging**<br>Deliverers: Embed implementation plans into existing practice	"[Having] the supports and the tools to do it easily so that there are no barriers…that sort of thing is really important." (M11)<br><br>"The best way to make this stuff work is to make people aware it's there and help them understand how to use it, but then to embed it in their workflow so they don't forget." (M15)
	**Engaging**<br>Deliverers:<br>Endorsing benefits	"The best way to make this stuff work is to make people aware it's there and help them understand how to use it, but then to embed it in their workflow so they don't forget." (M15)<br><br>"If you can bring it back to that value and that it's improving the work that they're doing and outcomes for the families, then that's going to be a huge benefit." (M13)
	**Engaging**<br>Recipients: endorsing intended benefits	"It highlights the importance if they're sitting together doing it…I also think it then shows the commitment of the IPC staff member too." (M13)<br><br>"I think it's education around why would you do this? Because this a different way of receiving information about parenting issues that a lot of people struggle with." (SP10)
	**Engaging**<br>Recipients:<br>encouraging ongoing engagement	"I think what I've learnt is that sometimes being just a bit too ambiguous about things doesn't get things done" (SP19)<br><br>"I don't think it's ever a case of forcing people to finish things…but you don't get the benefit unless you actually finish the thing. So, if you're going to actually draw their attention to it you may as well help them go through the program to maximise the potential of the benefit." (M15)
	**Reflecting and evaluating**<br>Protecting time for deliverers and	"It's about being able to take stock and reflect." (SP9)

	recipients to reflect and evaluate	"Workers are busy, they have to go to team meetings, so they're all going to be there, you'll have their attention, and I just think it really helps." (SP10)<br><br>"The end user voice…would be really important as we might be able to tailor it or pivot it, in a way that we think is most appropriate if it's not hitting the mark for some cohorts but it is for others. Having all of that information is going to be really quite critical to the success of it." (M12)

CFIR Barrier Results and Exemplar Quotes

**CFIR Domain**	**CFIR Construct**<br>Study-specific barrier theme	**Exemplar quotes**
Innovation	**Relative advantage**<br>Digital literacy and access	"Families who are struggling with low socioeconomic… or maybe there's a million reasons why they might not have access to that technology." (SP17)<br><br>"I think for the client a barrier could be technology for some of them." (SP1)
	**Design quality and packaging**<br>Use of sporting commentary genre	"For families who don't have that in their culture…[they] might find the format maybe a bit confusing. They might not understand why they're presenting in this commentator standpoint." (SP17)<br><br>"Podcasts are very sport orientated at the moment and my big thing is whether we lose families who aren't interested in sport or don't like that commentary style." (SP1)
Inner setting	**Work infrastructure**<br>Existing challenges to service functioning	"Adding something more would be a stretch" (SP14)<br><br>"It's not about positive or negative intent, it is simply about how crowded their… attention space is for options when it comes to clients." (M15)

		"To introduce something else on top of that now would probably feel a little bit overwhelming and a little bit complex." (SP8)
	**Compatibility**<br>Time needed for sensitive parenting work	"Parenting issues with families, those conversations typically can be quite lengthy…one issue could take up the whole session." (SP10)<br><br>"I don't think those questions would be a five minute conversation, I think 15 minutes would probably be more appropriate." (SP19)
	**Relative priority**<br>Prioritising complex social needs	"The barrier might be just if our client cohort had more complex needs." (M11)<br><br>"It's not going to be suitable for every family because there's going to be some more basic fundamental needs in terms of material necessities, like crises, like housing insecurity. Things like that are going to mean that it's going to be difficult for them to prioritise." (SP9)
	**Incentives and rewards**<br>Training fatigue	"Everyone is so busy and we've had to implement so many new things over the last few years…there's a bit of that worker fatigue coming in…even though the program itself is quite simple." (SP1)<br><br>"There can often procedurally be a lot more stuff for them to do…they'd be sort of saying, 'Well, you're giving me one more thing to do.'" (M11)
	**Available resources**<br>Timeliness of procuring digital resources	"I'm not in charge of that, so I'm not sure how viable that is." (SP17)<br><br>"We don't really have the resources as such to go purchasing…that would be something we would have to request through [management]." (SP22)
Implementation process	**Planning**<br>Unclear outcomes and feedback	"I think a barrier could be if it's unclear how outcomes might be measured or fed back…People might not be clear of what the value of it is." (M12)

		"If it costs us money to implement [digital] solutions but we can't stat the time, then we don't get the benefits of being able to increase the capacity of our human resources because we can't actually document [their] value." (M15)
	**Engaging**<br>Lack of translated versions for engaging recipients from CALD backgrounds	"We do have a lot of people who don't have English as their first language. Or aren't able to read English. I think some translated resources would be good." (M13)<br><br>"There will always be accessibility issues, unless it can be translated." (SP8)
	**Reflecting and evaluating**<br>Difficulty evaluating fidelity	"So that it is PaRK-Lite that we're actually implementing" (SP14)<br><br>"I also think it's important to keep it within the structuring evidence of the program itself because you can talk about lots of different parenting stuff but it's important to stay grounded in the evidence base of the program and what you're trying to achieve." (SP9)
Outer setting	**Partnerships and connections**<br>Momentum and relevance	"You don't have an end product, but that capacity to be able to brainstorm to see how they could be using [it]" (M24)<br><br>"When you're doing on the ground stuff…you're not really thinking about research and co-designing…it's just almost a foreign world." (M24)
